# Environmental factors and habitat use influence body condition of individuals in a species at risk, the grizzly bear

**DOI:** 10.1093/conphys/cou043

**Published:** 2014-10-03

**Authors:** Mathieu L. Bourbonnais, Trisalyn A. Nelson, Marc R. L. Cattet, Chris T. Darimont, Gordon B. Stenhouse, David M. Janz

**Affiliations:** 1 Spatial Pattern Analysis and Research Laboratory, Department of Geography, University of Victoria, Victoria, British Columbia, Canada V8W 3R4; 2 Canadian Wildlife Health Cooperative, Western College of Veterinary Medicine, University of Saskatchewan, Saskatoon, Saskatchewan, Canada S7N 5B4; 3 Applied Conservation Science Laboratory, Department of Geography, University of Victoria, Victoria, British Columbia, Canada V8W 3R4; 4 Foothills Research Institute, Hinton, Alberta, Canada T7V 1X6; 5 Department of Veterinary Biomedical Sciences, Western College of Veterinary Medicine, University of Saskatchewan, Saskatoon, Saskatchewan, Canada S7N 5B4

**Keywords:** Body condition, disturbance, grizzly bear, habitat, habitat net-energy demand, hair cortisol concentration

## Abstract

Metrics used to quantify the condition or physiological states of individuals provide proactive mechanisms for understanding population dynamics in the context of environmental factors. Our study examined how anthropogenic disturbance, habitat characteristics and hair cortisol concentrations interpreted as a sex-specific indicator of potential habitat net-energy demand affect the body condition of grizzly bears (*n* = 163) in a threatened population in Alberta, Canada. We quantified environmental variables by modelling spatial patterns of individual habitat use based on global positioning system telemetry data. After controlling for gender, age and capture effects, we assessed the influence of biological and environmental variables on body condition using linear mixed-effects models in an information theoretical approach. Our strongest model suggested that body condition was improved when patterns of habitat use included greater vegetation productivity, increased influence of forest harvest blocks and oil and gas well sites, and a higher percentage of regenerating and coniferous forest. However, body condition was negatively affected by habitat use in close proximity to roads and in areas where potential energetic demands were high. Poor body condition was also associated with increased selection of parks and protected areas and greater seasonal vegetation productivity. Adult females, females with cubs-of-year, juvenile females and juvenile males were in poorer body condition compared with adult males, suggesting that intra-specific competition and differences in habitat use based on gender and age may influence body condition dynamics. Habitat net-energy demand also tended to be higher in areas used by females which, combined with observed trends in body condition, could affect reproductive success in this threatened population. Our results highlight the importance of considering spatiotemporal variability in environmental factors and habitat use when assessing the body condition of individuals. Long-term and large-scale monitoring of the physiological state of individuals provides a more comprehensive approach to support management and conservation of species at risk.

## Abstract (precis)

We examined how environmental factors affected the body condition of grizzly bears in Alberta, Canada. Individual body condition varied significantly dependent on gender, age, human disturbance, vegetation productivity, and potential energetic demands. Our findings are important for understanding how body condition dynamics may influence recovery in this threatened population

## Introduction

Understanding how environmental factors, including anthropogenic activities and habitat characteristics, influence the health of wild animals is an increasingly important focus of wildlife research, with the potential to make major contributions to management and conservation efforts. While landscape fragmentation, habitat degradation and habitat loss have been linked to changes in species' distributions and population declines (Bender *et al.*, 1998; Fahrig, 2003), distributional changes are generally observed only once declines have begun, making conservation efforts difficult or ineffective (Wikelski and Cooke, 2006; Ellis *et al.*, 2012). Metrics to quantify the health or physiological state of individuals may provide a proactive mechanism for understanding how anthropogenic activities and habitat characteristics influence population dynamics.

Body condition is commonly used to assess the health of animals because it provides an estimate of fat reserves and the nutritional state of individuals (Jakob *et al.*, 1996). The body condition of an animal influences its reproductive performance (Cameron *et al.*, 1993; Guinet *et al.*, 1998; Robbins *et al.*, 2012), its ability to withstand disease and pathogens (Møller *et al.*, 1998), its vulnerability to predation (Murray, 2002) and its ability to survive periods of food scarcity (Millar and Hickling, 1990; Verrier *et al.*, 2011). Anthropogenic or natural factors that disrupt the use of important habitat and resources or limit the availability of food may have negative effects on the body condition of individuals (Delgiudice *et al.*, 2001; Rode *et al.*, 2006; Couturier *et al.*, 2009; Parker *et al.*, 2009). Ultimately, adverse effects on the body condition of individuals can impact the long-term persistence of threatened or endangered populations (Stevenson and Woods, 2006; Ellis *et al.*, 2012).

There is also growing recognition that stress in wild vertebrates resulting from continued or frequent exposure to noxious external stimuli may adversely affect the health of individuals (Wingfield *et al.*, 1998; Romero, 2004; Reeder and Kramer, 2005; Wikelski and Cooke, 2006). Vertebrates respond to external stressors through activation of the hypothalamic–pituitary–adrenal axis, which releases glucocorticoids into the blood circulation (Reeder and Kramer, 2005). Transient increases in circulating levels of glucocorticoid hormones allow the organism to respond to short-term stressors, with the goal of quickly re-establishing homeostasis (McEwen and Wingfield, 2003). However, high circulating levels of glucocorticoids for prolonged periods have been linked to decreased growth and reproductive capacity in animals (Wingfield and Sapolsky, 2003; Charbonnel *et al.*, 2008), diminished immune system performance (Acevedo-Whitehouse and Duffus, 2009; Bonier *et al.*, 2009a; Martin, 2009) and increased susceptibility to disease (Korte *et al.*, 2005; Acevedo-Whitehouse and Duffus, 2009).

The ability to quantify body condition and glucocorticoid levels in free-ranging animals provides opportunities to examine relationships between landscape conditions and the health of individuals. While a number of methods exist to estimate body condition from field measurements (see Jakob *et al.*, 1996; Green, 2001; Schulte-Hostedde *et al.*, 2005; Stevenson and Woods, 2006 for a discussion of body condition indices), the most frequently employed are residuals (both unstandardized and standardized) from an ordinary least-squares regression of body mass over body length that correlate with structural size (Schulte-Hostedde *et al.*, 2005). Stress in vertebrates is generally quantified by measuring glucocorticoid biomarkers, including cortisol levels, obtained from faeces, blood, saliva or hair samples (Macbeth *et al.*, 2010; Sheriff *et al.*, 2011). However, distinguishing baseline cortisol levels essential in energy regulation from cortisol levels that constitute a long-term stress response (i.e. allostatic overload) is difficult because repeated measures from individuals are required and baseline levels may vary seasonally or as an animal habituates to supposed stressors (Busch and Hayward, 2009; Martin, 2009). Despite this, a positive correlation between baseline cortisol levels and net energy demand, representing the difference between energy required and energy available (i.e. allostatic load), has been identified in a number of studies (reviewed by Busch and Hayward, 2009; Madliger and Love, 2014). However, to date few studies have integrated measures of body condition and glucocorticoid biomarkers with environmental data for large, far-ranging mammals (Ellis *et al.*, 2012).

Grizzly bears (*Ursus arctos*) occupy large home ranges and use diverse habitats during the course of the non-denning period. With fewer than 700 individuals remaining, grizzly bears were listed as Threatened in Alberta, Canada in 2010 (Nielsen *et al.*, 2009). This species occupies parks and protected areas, as well as a landscape heavily impacted by anthropogenic disturbance in Alberta. Large-scale on-going industrial activities prevalent throughout their remaining range include forestry, oil and gas exploration, mining and agriculture, all of which are serviced by an extensive road network. Roads provide access for both industry and recreation, and contribute to human–bear conflict and high rates of grizzly bear mortality (McLellan *et al.*, 1999; Benn and Herrero, 2002; Nielsen *et al.*, 2004b).

Habitat selection by grizzly bears within a multi-use heterogeneous landscape is complex (Nielsen *et al.*, 2002). A number of studies have shown that grizzly bears in Alberta select areas associated with anthropogenic disturbance and edge habitats, such as roads, pipelines, forest harvest blocks and oil and gas well sites (Nielsen *et al.*, 2004a; Berland *et al.*, 2008; Graham *et al.*, 2010; Stewart *et al.*, 2013; Laberee *et al.*, 2014). While selection of anthropogenic features has been found to vary according to grizzly bear age and gender in Alberta (e.g. Berland *et al.*, 2008; Graham *et al.*, 2010; Stewart *et al.*, 2013), these patterns are in contrast to those in other regions, where grizzly bears avoid roads, suggesting a potential degree of habituation to the risk associated with human proximity in this population (Berger, 2007).

Patterns of grizzly bear habitat selection associated with anthropogenic features in Alberta are thought to be driven by the presence and abundance of foods associated with these disturbances (Nielsen *et al.*, 2004c; Roever *et al.*, 2008). As the diet of grizzly bears in Alberta is comprised primarily of herbaceous foods and fruits (Mowat and Heard, 2006; Munro *et al.*, 2006; Nielsen *et al.*, 2010), the distribution of, and access to, high-quality foraging sites in the context of human activities has important implications for the body condition of grizzly bears. For example, Cattet *et al.* (2002) found that grizzly bears outside high-elevation parks and protected areas were generally in better body condition compared with those found inside parks and protected areas, potentially due to greater food availability. Likewise, Bourbonnais *et al.* (2013) observed that predicted hair cortisol concentrations, a potential indicator of long-term stress, in female grizzly bears were consistently lower in areas with increased anthropogenic disturbance. These patterns may reflect greater food availability associated with anthropogenic disturbance features or habituation to potentially stressful landscapes (Martin, 2009).

Although food sources associated with anthropogenic disturbances offer an opportunity for bears to improve body condition, the benefits may be outweighed by the high mortality risk associated with these habitats (Frid and Dill, 2002; Nielsen *et al.*, 2004b, 2008). Furthermore, the energetic demands associated with anthropogenic features such as roads may be higher, because bears that use habitats in close proximity to roads may be more vigilant (Frid and Dill, 2002) or more likely to engage in a costly flight response (McLellan and Shackleton, 1988; Gibeau *et al.*, 2002). These activities require energy and, accordingly, may influence body condition. Given the threatened status of grizzly bears in Alberta, combined with their low population densities (Proctor *et al.*, 2012), low reproductive rates (Proctor *et al.*, 2012) and high mortality rates (McLellan *et al.*, 1999; Benn and Herrero, 2002; Nielsen *et al.*, 2004b), a more thorough examination of how spatiotemporal variability in environmental factors influences grizzly bear body condition should promote conservation efforts.

In this analysis, our goal is to explore how anthropogenic disturbance, habitat characteristics and energetic demands associated with a spatial index of hair cortisol concentrations (HCC) influence the body condition of individual grizzly bears quantified using a body condition index (BCI; Cattet *et al.*, 2002). Although HCC has been advocated as a potential biomarker of long-term stress in grizzly bears (Macbeth *et al.*, 2010), we do not yet know what HCC levels constitute allostatic overload vs. allostatic load in this species. Thus, we are using predicted HCC levels (as determined by Bourbonnais *et al.*, 2013) in this study as an integrated, sex-specific index of habitat net-energy demand. We suggest that index values are higher in habitat areas where environmental factors (e.g. reduced food abundance and/or quality, greater topographic complexity and increased human activity) are likely to result in greater allostatic load for resident bears (Bourbonnais *et al.*, 2013; Bryan *et al.*, 2013, 2014). To account for the influence of environmental covariates, we use global positioning system (GPS) telemetry data to characterize habitat use. We also consider biological factors and capture effects that are known to influence grizzly bear body condition (Cattet *et al.*, 2008; Boulanger *et al.*, 2013; Nielsen *et al.*, 2013a).

## Materials and methods

### Study area

**Figure 1:**
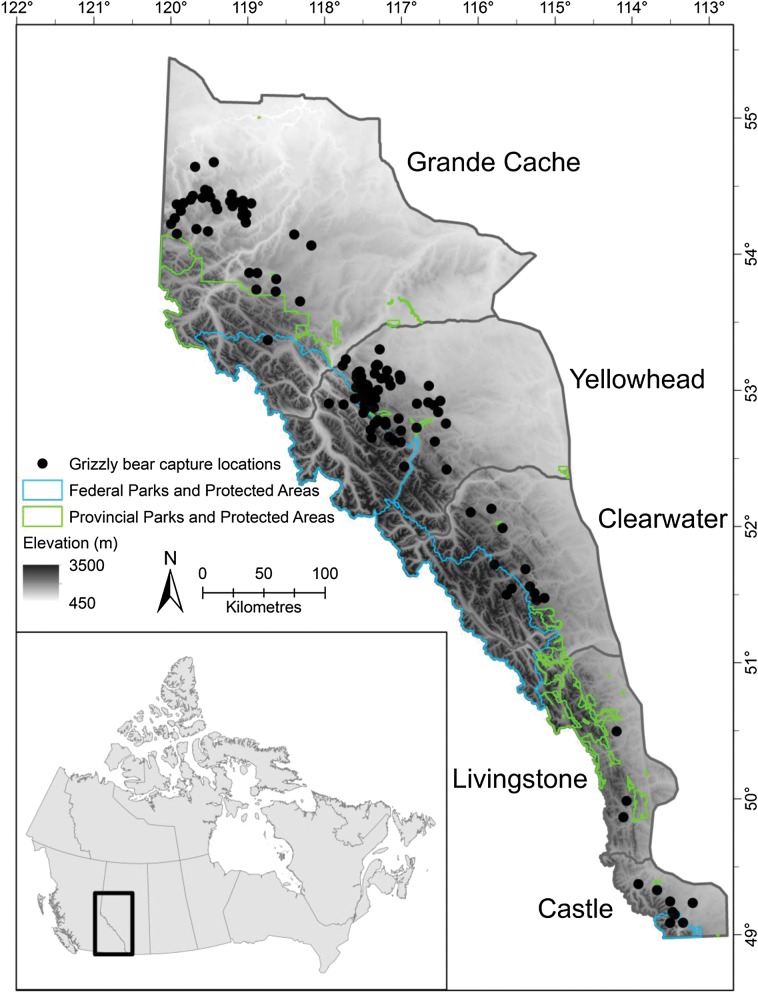
Grizzly bear capture locations in Alberta, Canada. A total of 163 grizzly bears were captured from 1999 to 2010 between April and October using a combination of leg-hold snares, culvert traps and remote drug delivery from a helicopter. Note that multiple bears were captured at specific culvert trap locations during the study period. Body condition was determined at the time of capture, and each bear was fitted with a GPS radiocollar to allow assessment of spatial patterns of habitat use. The five bear management units represent an area of nearly 111 000 km^2^.

Our study area was located in the Grande Cache, Yellowhead, Clearwater, Livingstone and Castle grizzly bear management units in Alberta, Canada, which have a combined area of nearly 111 000 km^2^ (Fig. 1). Elevation in the study area ranges from 450 to 3500 m and increases from east to west. Habitat types include alpine and sub-alpine ecosystems, mixed-wood forests and wet-meadow complexes (Stenhouse *et al.*, 2005). Mean temperature ranges from 12°C in the summer to −7.5°C in the winter, and mean annual precipitation is 450–800 mm. An extensive road network built to service industrial activities also provides recreational access for a variety of activities, including hunting, fishing, trapping, hiking and trail-riding with all-terrain vehicles and snowmobiles. A network of federal and provincial parks and protected areas, which prohibit resource-extraction activities, are found throughout the study area.

### Bear captures and body condition index

We assessed the body condition of 163 grizzly bears (*n* = 69 males and 94 females) captured between 1999 and 2010. Captures occurred from April until October in order to account for potential changes in body condition dynamics over the entirety of the non-denning period, although the majority of captures were made between April and June. Bears were captured by the Foothills Research Institute Grizzly Bear Project using a combination of leg-hold snares, culvert traps and remote drug delivery from a helicopter. Captures followed protocols accepted by the Canadian Council of Animal Care for the safe handling of bears (University of Saskatchewan Committee on Animal Care and Supply Protocol number 20010016). We fitted a VHF ear-tag transmitter (Advanced Telemetry Systems) and a GPS radiocollar from either Televilt Simplex, Tellus (Followit; Lindesberg, Sweden) or Advanced Telemetry Systems (Isanti, MN, USA) to each bear. The GPS-based locations were obtained at 4 h intervals prior to 2004 and at 1–2 h intervals after 2004.

We determined the age of each bear using microscopic analysis of a premolar section (Stoneberg and Jonkel, 1966). For each individual, we recorded the gender, reproductive status (e.g. with or without cubs, and the age of cubs), and the number of times the bear had been captured previously (Cattet *et al.*, 2008; Boulanger *et al.*, 2013; Nielsen *et al.*, 2013a). We classified individuals as adult (>5 years old) males (*n* = 47) or females (*n* = 55), juvenile (3–5 years old) males (*n* = 22) or females (*n* = 22), and females with cubs-of-year (COY; *n* = 17). We distinguished females with COY from females with older cubs (which were grouped with adult females) because females with COY have greater energetic requirements (Farley and Robbins, 1995) and tend to have smaller home ranges (Dahle and Swenson, 2003; Smulders *et al.*, 2012). We weighed and measured bears using a load-scale and a tape stretched from the tip of the nose to the last tail vertebrae. We used weight and length measurements from individuals captured in the field to obtain BCI values that represent the standardized residuals from a linear regression of log-transformed total body mass and straight-line body length (Cattet *et al.*, 2002). The BCI values used in this analysis ranged from −3 to +3.

### Grizzly bear habitat use

We characterized grizzly bear habitat use using GPS-based positional data from the period (30–60 days) following the capture of each individual. The 30- to 60-day post-capture period was selected to quantify habitat use in order to avoid potential capture effects on grizzly bear movement rates (Cattet *et al.*, 2008). We used fixed-kernel density estimation to calculate utilization distributions (package adehabitatHR in R version 2.15.0; Calenge, 2013) from the GPS data with a bandwidth defined by least-squares cross-validation (Worton, 1989). We contoured the utilization distributions at the 95th percentile isopleth in order to represent the area used by each individual. Areas of habitat use, which represent the spatial unit of analysis, were used to summarize anthropogenic, habitat and habitat net-energy demand variables. To assess the similarity between post-capture habitat use and potential pre-capture habitat use, we compared the areal extent of the area of habitat use of 50 bears captured in the spring with the area of habitat use for the same animal from the preceding autumn based on GPS data for 30 days prior to den entry. The coefficient of variation between the areal extent of pre-capture aumtumn habitat use and post-capture spring habitat use was 38% for the 50 bears considered. Furthermore, the mean change in the habitat-use area centroid co-ordinates of the pre-capture autumn and post-capture spring periods was minimal (∼4 km in both the Easting and Northing directions). As a result, the post-capture characterization of grizzly bear habitat use is a reasonable approximation of the area used prior to capture, giving us confidence that BCI values can be related to environmental variables based on post-capture GPS telemetry data.

### Covariate data

We modelled and temporally matched covariate data representing anthropogenic features, habitat characteristics and habitat net-energy demand with grizzly bear habitat-use areas in a Geographic Information System (GIS; see Table 1 for rationale and data sources). Anthropogenic disturbance features that we considered included all-weather roads, oil and gas well sites, seismic lines, power lines, pipelines and forest harvest blocks. We characterized the localized influence of roads and oil and gas well sites using an exponential distance decay function, *e*^*−ad*^, where *d* is the distance in metres to the feature and *a* is fixed at 0.002 (Nielsen *et al.*, 2009). We represented secondary linear features (e.g. seismic lines, power lines and pipelines), which provide access to grizzly bear habitat and contribute to landscape fragmentation (Linke *et al.*, 2005; Stewart *et al.*, 2013), as a cumulative linear density (in kilometres per square kilometre) within each habitat-use area. We represented forest harvest blocks based on the areal density (in kilometres per square kilometre) of these features within habitat-use areas. We quantified the influence of parks and protected areas, which represent a noted contrast in land use compared with the surrounding landscape, based on the areal percentage of habitat use that occurred within parks and protected areas.

**Table 1:** The biological, anthropogenic and habitat-related covariates considered to explain body condition of grizzly bears in Alberta, Canada

Covariate	Rationale	References	Data source
Reproductive class	Based on gender, age and presence of cub(s)-of-year, which influence habitat selection patterns and energetic demands, individuals were classified as adult males or females (>5 years old), juvenile males or females (2–5 years old) or adult females with cub(s)-of-year	Boulanger *et al.* (2013) Nielsen *et al.* (2013a)	Grizzly bear capture data
Number of previous captures	Multiple handlings may adversely influence body condition	Cattet *et al.* (2008)	
Capture date (Julian date)	Seasonal changes in food availability and habitat selection during the non-denning period may influence body condition	McLellan (2011)	
Index of habitat net-energy demand	Factors related to anthropogenic disturbance and habitat characteristics influence predicted hair cortisol concentrations in grizzly bears. Predicted hair cortisol concentration values are interpreted as a sex-specific indicator of net-energy demand	Macbeth *et al.* (2010) Bourbonnais *et al.* (2013) Bryan *et al.* (2013)	Bourbonnais *et al.* (2013)
Roads (distance decay)	Provide human access to grizzly bear habitat; contribute to landscape fragmentation; herbaceous foods are present in areas adjacent to roads	Munro *et al.* (2006) Berland *et al.* (2008) Roever *et al.* (2008) Graham *et al.* (2010)	AESRD; FRIGBP; Landsat 5 TM; Landsat 7 ETM +
Oil and gas well sites (distance decay)	Localized areas of human activity; create forest edges and contribute to landscape fragmentation	Laberee *et al.* (2014)	
Density of secondary linear features (km/km^2^)	Seismic lines, power lines and pipelines create forest edges and contribute to landscape fragmentation and provide access to grizzly bear habitat	Linke *et al.* (2005) Stewart *et al.* (2013)	
Density of forest harvest blocks (km/km^2^)	Disturbance features associated with presence and abundance of herbaceous foods	Nielsen *et al.* (2004a, c) Munro *et al.* (2006) Berland *et al.* (2008)	
Percentage of parks and protected areas	Considered core refugia and represent a marked contrast in land use compared with the surrounding industrialized landscape	Gibeau *et al.* (2002)	
Elevation (variation)	Influences vegetation composition, human access and potential habitat net-energy demand	Nielsen *et al.* (2004b, c) Bourbonnais *et al.* (2013)	Landsat 5 TM; Landsat 7 ETM+; DEM
Crown closure (variation)	Influences understory vegetation abundance and growth of herbaceous foods	Franklin *et al.* (2002, 2003) Nielsen *et al.* (2013a)	
Percentage of conifer tree cover	Characterization of forest species distribution and correlated with berry abundance	Franklin *et al.* (2002, 2003)	
Percentage of mixed and broadleaf tree cover	Influences distribution of herbaceous foods and correlated with presence of ungulates	Nielsen *et al.* (2010) Stewart *et al.* (2013)	
Percentage of regenerating forest	Regenerating forests have greater availability of herbaceous foods	Nielsen *et al.* (2004c, 2010)	
Percentage of shrub and herbaceous landcover	Correlated with availability of herbaceous foods and berries	Franklin *et al.* (2002, 2003)	
Forest age	Younger seral forests have a greater abundance of herbaceous foods	Nielsen *et al.* (2004c, 2010)	
Vegetation productivity	Total vegetation productivity (cumulative greenness) influences availability of herbaceous foods	Coops *et al.* (2008) Fontana *et al.* (2012)	AVHRR DHI
Vegetation seasonality	Seasonal variability (coefficient of variation) in vegetation productivity influences timing and availability of herbaceous foods	Coops *et al.* (2008) Fontana *et al.* (2012)	

Abbreviations: AESRD, Alberta Environment and Sustainable Resource Development; AVHRR, Advanced Very High Resolution Radiometer; DEM, digital elevation model; DHI, Dynamic Habitat Index; ETM+, Enhance Thematic Mapper Plus; FRIGBP, Foothills Research Institute Grizzly Bear Project; TM, Thematic Mapper.

We selected habitat variables that characterized forest conditions, landcover, topography and vegetation productivity, and which represented proxies of potential food availability (see Table 1 for rationale and data sources). We quantified forest composition and structure within habitat-use areas based on the variance in crown closure, the percentage of coniferous forest, the percentage of mixed and broadleaf tree cover, the percentage of regenerating forest, the mean forest age and the percentage of shrub and herb landcover (Franklin *et al.*, 2002, 2003). We characterized topography associated with habitat use based on the variance in elevation. We used the cumulative greenness (total annual productivity) and coefficient of variation (seasonality) indices from the Dynamic Habitat Index (DHI) to quantify vegetation productivity within the habitat-use area of each bear (Coops *et al.*, 2008). The two DHI indices are obtained by summarizing annual trends in monthly images of the fraction of photosynthetically active radiation derived from Advanced Very High Resolution Radiometer reflectance values (Fontana *et al.*, 2012). We used a data product representing a spatial index of predicted HCC levels to characterize the potential habitat net-energy demand associated with grizzly bear habitat-use patterns (see Bourbonnais *et al.*, 2013 for details). Stratified by gender, we calculated a habitat net-energy demand index value for each habitat-use area to represent the potential energetic demands associated with the habitat characteristics of the area.

### Statistical analyses

We used linear mixed-effects models (package nlme in R version 2.15.0; Pinheiro, 2013) to examine the relationships between the dependent grizzly bear BCI response and the independent biological, anthropogenic, habitat and habitat net-energy demand index variables (Pinheiro and Bates, 2000). Continuous independent variables were centred and scaled due to the range in values and to aid interpretation of regression coefficients (Schielzeth, 2010). We limited collinearity and redundancy in covariates by excluding those with a Pearson correlation coefficient ≥0.6 and a variance inflation factor ≥5. We found that elevation was strongly correlated with a number of covariates, including the DHI variables, crown closure, roads and the percentage of parks and protected areas. As a result, we excluded elevation from the models because the DHI metrics adequately represented the variability in vegetation productivity resulting from elevation gradients.

We considered separate anthropogenic and habitat linear mixed-effects models, as well as a global model combining covariates from the anthropogenic and habitat models. We assessed the support for the three models considered using Akaike weights (*w*_*i*_) based on Akaike information criterion (AIC) values (Burnham and Anderson, 2002). We controlled for biological and capture effects on body condition by including in each of the models the reproductive class, the number of previous captures and the capture date. As habitat characteristics and anthropogenic activities influence habitat net-energy demand (Bourbonnais *et al.*, 2013), we included values from this index in the anthropogenic, habitat and global models. In order to examine the influence of habitat net-energy demand on body condition further, we used a factorial ANOVA to compare potential energetic demands associated with habitat use in each of the five reproductive classes considered.

Covariates representing biology, anthropogenic disturbance, forest characteristics, vegetation productivity and habitat net-energy demand were included as fixed effects, with a unique identifier for each bear as the random effect in the respective models. As suggested by Zuur *et al.* (2009), we refitted the models using restricted maximum likelihood estimation to limit bias in the regression coefficients. We found no evidence of correlation of predictor variables in the final anthropogenic, habitat and global models, and within-group residuals appeared to be normally distributed (Pinheiro and Bates, 2000; Zuur *et al.*, 2009). We assessed the normality of the random effects by plotting the best linear unbiased estimators for each model (Pinheiro and Bates, 2000). These were acceptable for all three of the models considered. We quantified the variance explained by fixed effects in each model using a marginal *r*^2^ and the cumulative variance explained by fixed and random effects using a conditional *r*^2^ (Nakagawa and Schielzeth, 2013).

## Results

The global linear mixed-effects model including biological, habitat net-energy demand, anthropogenic and habitat-related covariates had the strongest support (*w*_*i*_ = 0.92) among the models considered (Table 2). We found limited support for candidate models combining biological covariates and habitat net-energy demand with habitat covariates (*w*_*i*_ = 0.10) and anthropogenic covariates (*w*_*i*_ = 0.01), respectively. The variance explained was also higher for the global linear mixed-effects model (marginal *r*^2^ = 0.44; conditional *r*^2^ = 0.56) compared with the habitat model (marginal *r*^2^ = 0.37; conditional *r*^2^ = 0.53) and the anthropogenic model (marginal *r*^2^ = 0.34; conditional *r*^2^ = 0.47; Table 2).

**Table 2:** Model selection results comparing anthropogenic, habitat and global linear mixed-effects candidate models considered to explain grizzly bear body condition in Alberta, Canada

Model (*i*)	Candidate model	AIC	ΔAIC	*w*_*i*_	*r*^2^
Global	Anthropogenic model + habitat model	411.6	0.00	0.92	0.44 (0.56)
Habitat	Reproductive class + capture date + number of previous captures + habitat net-energy demand + crown closure (variance) + percentage of conifer + percentage of mixed and broadleaf tree cover + percentage of regenerating forest + percentage of shrub and herbaceous landcover + forest age + vegetation productivity + vegetation seasonality	416.7	5.13	0.10	0.37 (0.53)
Anthropogenic	Reproductive class + capture date + number of previous captures + habitat net-energy demand + density of forest harvest blocks + density of secondary linear features + roads (distance decay) + well sites (distance decay) + percentage of parks and protected areas	419.9	8.34	0.01	0.34 (0.47)

Abbreviations: AIC, Akaike information criterion; ΔAIC, difference in Akaike information criterion between the most supported model and the given model; the marginal *r*^2^ and conditional (*r*^2^) for each candidate model; and *w*_*i*_, weight of evidence for the *i*th model.

Influential covariates related to anthropogenic factors in the global model included the density of forest harvest blocks, roads (distance decay), the percentage of parks and protected areas, and oil and gas well sites (distance decay; Table 3). We found that grizzly bear body condition was improved when spatial patterns of habitat use included increasing densities of forest harvest blocks (*P* = 0.022) and decreasing distance (decay) to well sites (*P* = 0.099). Grizzly bear body condition was poorer when spatial patterns of habitat use were characterized by lower distances (decay) to roads (*P* = 0.043) and a greater percentage of parks and protected areas (*P* = 0.065). The density of secondary linear features (*P* = 0.424) did not influence grizzly bear body condition.

**Table 3:** Parameter estimates from the global linear mixed-effects model explaining grizzly bear body condition in Alberta, Canada

Parameters	β	±SE	d.f.	*t* value	*P* value
Intercept	0.03	0.30	111	0.10	0.92
Female with cub(s)-of-year	−1.45	0.27	31	−5.38	**<0.001**
Adult female	−1.20	0.20	31	−6.04	**<0.001**
Juvenile female	−1.32	0.23	31	−5.80	**<0.001**
Juvenile male	−0.75	0.22	31	−3.33	**0.002**
Capture date	0.01	0.00	31	2.81	**0.009**
Number of previous captures	−0.12	0.06	31	−1.94	**0.062**
Habitat net-energy demand	−0.23	0.10	31	−1.85	**0.073**
Density of forest harvest blocks	0.42	0.17	31	2.42	**0.022**
Density of secondary linear features	−0.13	0.16	31	−0.81	0.424
Distance decay to roads	−0.38	0.18	31	−2.11	**0.043**
Distance decay to well sites	0.33	0.20	31	1.70	**0.099**
Percentage of parks and protected areas	−0.18	0.10	31	−1.91	**0.065**
Crown closure (variance)	−0.16	0.09	31	−1.92	**0.064**
Percentage of conifer	0.29	0.12	31	2.40	**0.023**
Percentage of regenerating forest	0.17	0.09	31	1.91	**0.065**
Percentage of mixed and broadleaf tree cover	0.13	0.12	31	1.16	0.256
Percentage of shrub and herbaceous landcover	−0.00	0.09	31	−0.04	0.968
Forest age	−0.01	0.14	31	−0.10	0.923
Vegetation productivity (DHI)	0.66	0.23	31	2.86	**0.008**
Vegetation seasonality (DHI)	−0.40	0.15	31	−2.57	**0.015**

The table shows parameter estimates (β), standard errors (±SE), degrees of freedom (d.f.), *t* values and parameter statistical significance (*P* values). Abbreviation: DHI, Dynamic Habitat Index. The model was refitted using restricted maximum likelihood estimation. Statistically significant parameters (*P* = 0.1) are indicated in bold.

The two DHI metrics, cumulative greenness (vegetation productivity; *P* = 0.008) and the coefficient of variation (vegetation seasonality; *P* = 0.015), had the greatest influence on grizzly bear body condition among covariates representing habitat characteristics. We found that bears whose spatial patterns of habitat use were characterized by increased vegetation productivity were in better body condition. Increased vegetation seasonality was associated with poorer body condition. Covariates related to forest composition and structure showed that an increased percentage of conifer (*P* = 0.023) and percentage of regenerating forest (*P* = 0.065) in areas of habitat use improved body condition. Conversely, increased variance in crown closure (*P* = 0.064) resulted in poorer grizzly bear body condition. The percentage of mixed and broadleaf tree cover (*P* = 0.256), the percentage of shrub and herbaceous landcover (*P* = 0.968) and forest age (*P* = 0.923) were not related to grizzly bear body condition.

**Figure 2:**
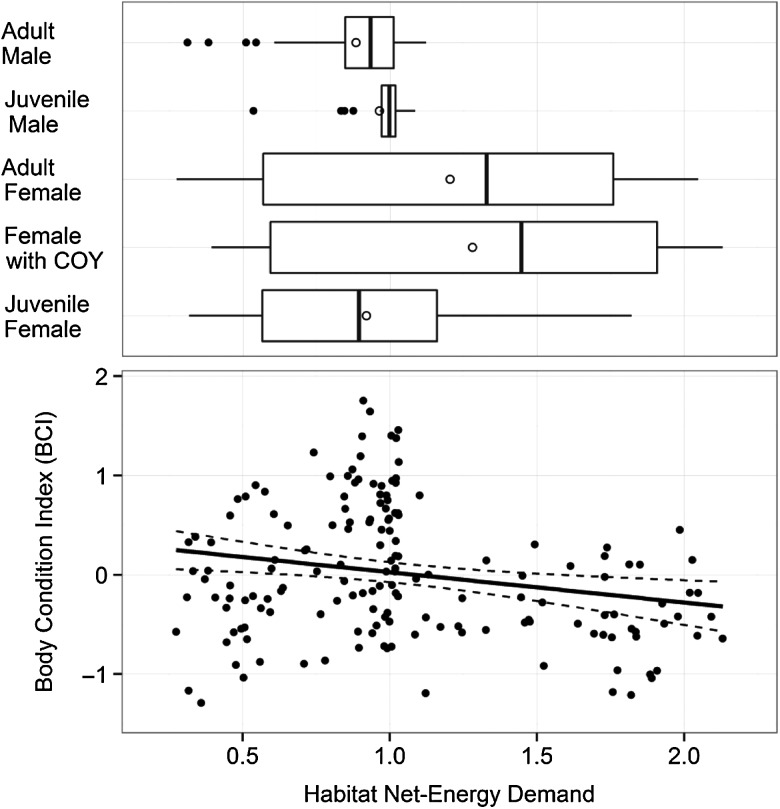
The observed association between body condition index (BCI), habitat net-energy demand and reproductive class for 163 grizzly bears in Alberta, Canada. The best-fit line in the lower plot is the estimate from the global linear mixed-effect model in Table 3 and the dashed lines are the 95% confidence bands. Marginal boxplots in the upper plot show the habitat net-energy demand values associated with each reproductive class. The boxes represent the median, 25th and 75th percentiles, the lines represent 1.5 times the interquartile range, the filled circles represent outliers and the open circles the mean habitat net-energy demand. Habitat net-energy demand values represent predicted hair cortisol concentrations associated with habitat characteristics (detailed by Bourbonnais *et al.*, 2013).

We found that spatial patterns of grizzly bear habitat use associated with increased habitat net-energy demand index values (*P* = 0.073) resulted in decreased body condition (Fig. 2). The factorial ANOVA showed that habitat net-energy demand in habitat-use areas of the five reproductive classes we considered differed significantly (*F* = 4.87, *P* < 0.001). Observed potential habitat net-energy demand was lower for adult males (mean habitat net-energy demand, 0.88 ± 0.03) compared with juvenile females (mean habitat net-energy demand, 0.92 ± 0.09), juvenile males (mean habitat net-energy demand, 0.96 ± 0.02), adult females (mean habitat net-energy demand, 1.20 ± 0.08) and females with COY (mean habitat net-energy demand, 1.27 ± 0.16; Fig. 2). Among covariates related to field-capture data, we found that an increasing number of previous captures (*P* = 0.062) resulted in poorer body condition, while body condition improved as the date of capture (*P* = 0.009) occurred later in the non-denning period. Finally, we found that factors related to grizzly bear reproductive class (*P* < 0.001), which classified individuals based on gender, age and the presence of COY, had the strongest effect in the global model, because females with COY (mean BCI, −0.43 ± 0.11), juvenile females (mean BCI, −0.48 ± 0.09), adult females (mean BCI, −0.28 ± 0.06) and juvenile males (mean BCI, −0.11 ± 0.07) were in significantly poorer body condition compared with adult males (mean BCI, 0.76 ± 0.06).

## Discussion

Our study has demonstrated how factors related to anthropogenic disturbance, habitat characteristics, potential habitat net-energy demand and biology combine to influence the body condition of grizzly bears in a threatened population in Alberta, Canada. Grizzly bears in Alberta occupy a multi-use landscape, resulting in complex spatial associations among anthropogenic disturbance features, habitat characteristics and body condition. For example, forest harvest blocks and oil and gas well sites that create access to herbaceous foods (Nielsen *et al.*, 2004a, c; Laberee *et al.*, 2014) provided gains in body condition. Likewise, regenerating forest conditions, which are generally associated with anthropogenic rather than natural disturbances in Alberta, allowed bears to improve body condition. However, bears whose habitat-use patterns were in closer proximity to roads, the majority of which are built to service forest and oil and gas industries, were in poorer body condition.

Road densities and selection of roadside habitats are important factors influencing survival in grizzly bears (McLellan *et al.*, 1999; Benn and Herrero, 2002; Nielsen *et al.*, 2004b). In a recent study examining grizzly bear body condition and mortality, Boulanger *et al.* (2013) found that bears were in better body condition when road densities and variation in regenerating forest were higher, but they had a considerably higher mortality compared with bears in areas with lower road densities and less variation in regenerating forest. While we observed a negative relationship between roads and grizzly bear body condition, our study considered the localized influence of roads based on distance rather than density. Depending on spatial patterns of habitat use, an individual may occupy an area with low road densities and yet its general pattern of selection may occur in close proximity to roads. Regardless, high mortality rates associated with roads and regenerating forest conditions may negate any potential gains in body condition resulting from the use of disturbance features such as forest harvest blocks and well sites.

Despite low rates of grizzly bear mortality in parks and protected areas, as well as limited anthropogenic access and disturbance, individuals whose patterns of habitat use occurred predominantly in these areas were in poorer body condition. Many parks and protected areas in the region are located in mountainous terrain, with highly seasonal vegetation productivity and high variation in crown closure (e.g. transitions between alpine and forest), which negatively influenced body condition. The overall poor body condition of individuals that occupy seasonal environments, which may influence the timing of life-history events in grizzly bears (Ferguson and McLoughlin, 2000), may partly explain the low reproductive rates observed in parks and protected areas like Jasper and Banff National Park (Proctor *et al.*, 2012). In comparison, regions outside of parks and protected areas are characterized by higher overall vegetation productivity, allowing individuals to improve their body condition. While grizzly bears are omnivorous, they depend largely on herbaceous growth and berries to meet their nutritional needs in Alberta (Mowat and Heard, 2006; Munro *et al.*, 2006). As a result, total vegetation productivity and seasonality appear to influence body condition in grizzly bears in a manner similar to results observed in ungulates, where the timing and duration of vegetation onset influences the body mass and condition of individuals (Pettorelli *et al.*, 2007).

Our approach for determining pre-capture habitat use based on telemetry data from the 30- to 60-day post-capture period provided a reasonable estimate of the area used by each individual. However, this approach is a potential limitation of our study because post-capture GPS telemetry data may not completely reflect pre-capture habitat use and may partly account for a portion of the unexplained variance in BCI values. There are also a number of other notable biological and environmental factors which may influence body condition that we did not consider here. Many of the environmental covariates that we considered, including forest harvest block density, percentage of regenerating forest, and vegetation productivity and seasonality quantified using the DHI metrics, represent proxies for grizzly bear food availability and habitat quality rather than direct measures of nutritional condition. Incorporating more direct measures of individual nutritional condition and food availability using methods such as stable isotope analysis (e.g. Hilderbrand *et al.*, 1999; McLellan, 2011; Bryan *et al.*, 2013, 2014) or landscape-based food models (e.g. Nielsen *et al.*, 2010) could help to explain more of the individual variability in body condition.

Body condition during the months of April and May, which represented the majority of our data, was also highly variable within all five of the reproductive classes we considered. Some of the springtime variability in body condition is likely to result from biological and environmental factors related to the preceding non-denning and denning periods. For example, Nielsen *et al.* (2013a) found that springtime body mass and body length were influenced by climatic conditions from the previous summer and winter seasons. They also found that individual body condition was partly dependent on natal climatic conditions, representing a potential silver-spoon effect. Pre-denning body mass strongly influences the energetic reserves that allow individuals to meet demands while fasting during the winter (Hilderbrand *et al.*, 2000). Energetic demands while denning are especially high for lactating females with cubs, whose body mass loss is substantially higher compared with non-lactating individuals (Farley and Robbins, 1995). Consequently, consideration of habitat use, climate and biological factors such as body mass, body condition, or presence of cubs from the previous pre-denning and denning periods could account for increased variance in springtime body condition. However, including these factors would have substantially reduced our total sample size because it requires data for individuals over consecutive seasons.

Overall body condition increased as the date of capture occurred later in the summer and autumn in all the reproductive classes. This highlights the importance of considering body condition dynamics over the entirety of the non-denning period. Seasonal trends in body condition are likely to result from changes in habitat use and food availability, as well as an increased focus on improving condition before denning. Similar seasonal body condition dynamics have been observed in grizzly bear populations in the interior of British Columbia (McLellan, 2011) and are evident in other species that experience prolonged periods of fasting or nutritional deficits (Couturier *et al.*, 2009). Adult male grizzly bears also tended to be in better body condition compared with adult females, females with COY and juveniles of both sexes throughout the non-denning period. The reproductive classes we incorporated may reflect potential intra-specific social dynamics. A number of studies have demonstrated that grizzly bears exhibit sexual and competitive segregation in the selection of high-quality habitat related to gender, age and the presence of cubs (Rode *et al.*, 2001, 2006; Dahle *et al.*, 2006; Smulders *et al.*, 2012; Nielsen *et al.*, 2013b; Steyaert *et al.*, 2013). Furthermore, in a study of coastal black bears and grizzly bears, Bryan *et al.* (2014) suggested that the avoidance by black bears of habitat used by larger and more aggressive grizzly bears could result in less access to high-quality habitat and foods, explaining higher observed cortisol levels in black bears. Due to their larger size, adult males could also have a competitive advantage in the selection of high-quality habitat and more opportunity to increase their body condition compared with smaller females and juvenile grizzly bears.

Using a spatial index of predicted hair cortisol concentrations developed by Bourbonnais *et al.* (2013) as an indicator of sex-specific habitat net-energy demand, our study demonstrated that grizzly bear body condition was negatively affected by high potential energetic demands associated with habitat characteristics. This is in agreement with Macbeth *et al.* (2012) and Cattet *et al.* (2014), who found that body condition was inversely associated with hair cortisol concentrations in captured polar bears and grizzly bears, respectively. While glucocorticoids levels are commonly interpreted as an indicator of long-term stress, distinguishing baseline levels from those that constitute a stress response in free-ranging animals is difficult (Busch and Hayward, 2009; Boonstra, 2013; Cattet *et al.*, 2014; Madliger and Love, 2014). Glucocorticoids are important mediators of energy homeostasis and, as a result, healthy organisms will increase glucocorticoid release as part of their normal physiology (McEwen and Wingfield, 2003; Busch and Hayward, 2009; Boonstra, 2013). For example, elevated glucocorticoids serve a critical role in gluconeogenesis in grizzly bears that fast for prolonged periods during hibernation (Hellgren, 1998). However, considering the energetic demands associated with baseline glucocorticoid levels in organisms can provide a useful framework for considering the physiological impacts associated with environmental factors (Madliger and Love, 2014).

A number of studies have found that baseline cortisol levels in vertebrates increase in response to environmental factors resulting in decreased body condition (Kitaysky *et al.*, 1999; Suorsa *et al.*, 2003; Bonier *et al.*, 2009b). In grizzly bears, elevated cortisol levels have been associated with anthropogenic disturbance and environmental factors (Bourbonnais *et al.*, 2013), as well as the availability and accessibility of high-quality foods (Bryan *et al.*, 2013, 2014). Consequently, poor body condition in grizzly bears may in part be a result of habitat use where environmental or nutritional challenges require individuals to expend more energy. We acknowledge that energetic demands associated with habitat use will be likely to vary according to individual life history; for example, repeated exposure to external stressors can potentially lead to habituation and the suppression of a physiological response in an individual (Busch and Hayward, 2009; Martin, 2009). Yet consideration of potential energetic demands associated with habitat use dependent on broader scale biological classifications, such as sex or age class, can influence conservation efforts (Madliger and Love, 2014). Interestingly, we observed that potential energetic demands associated with habitat use were higher for females, particularly for adult females and females with COY, which had the poorest observed body condition and represent the reproductive demographic of this threatened population. Future studies should therefore consider how relationships between energetic demands and body condition could influence reproduction and survival in the species in order to assess population dynamics better (Fefferman and Romero, 2013).

### Conclusion

Spatial approaches are necessary in order to understand the complex interactions among animal physiology, behaviour and the environment. Grizzly bear body condition is known to be influenced by age and gender (Boulanger *et al.*, 2013; Nielsen *et al.,* 2013a). By considering spatial patterns of habitat use, we have shown that the body condition of grizzly bears is also dependent on the nature and intensity of anthropogenic disturbance, forest structure, vegetation productivity and seasonality and potential energetic demands associated with habitat characteristics. While bears may benefit from anthropogenic disturbance, gains in body condition may be offset by high mortality rates in areas with human access. Selection of habitat where environmental factors increase energetic demands may also negatively affect body condition. These costs may be compounded for the current and future reproductive demographic of this at-risk population because females with COY, adult females and juvenile females were in poorer body condition compared with adult males. Management efforts aimed at limiting human access may help reduce mortality rates and allow bears to benefit from gains in body condition associated with productive habitats. While our study focuses on spatial associations between grizzly bear body condition and environmental factors in Alberta, many interior grizzly bear populations in North America that rely primarily on herbaceous food sources face similar anthropogenic pressures. As a result, our methods and results may be of relevance for management efforts and studies in other systems.

This study further emphasizes the utility of physiology-based metrics, such as body condition indices and hair cortisol concentrations, for assessing environmental impacts on the health of individuals. As wildlife populations worldwide face increasing anthropogenic pressures, comprehensive approaches examining how spatial and temporal environmental variability influences the health of individuals are of increasing importance, especially for species at risk (Wikelski and Cooke, 2006; Ellis *et al.*, 2012). As demonstrated here, long-term and large-scale monitoring of body condition and glucocorticoids in at-risk or recovering populations can help identify the often unintended and sometimes unexpected consequences of anthropogenic habitat change (Cooke and O'Connor, 2010). By developing a more thorough understanding of the physiological responses of individuals to their environment, management and conservation efforts can be tailored to ensure the health of individuals and, as a result, the long-term viability of populations.

## Funding

This work was supported by the many funding partners of the Foothills Research Institute Grizzly Bear Program; Alberta Environment and Sustainable Resource Development; the Natural Sciences and Engineering Research Council of Canada; and the Yellowstone to Yukon Conservation Initiative Sarah Baker Memorial Fund.
